# Development of an immune-related prognostic biomarker for triple-negative breast cancer

**DOI:** 10.1080/07853890.2022.2067894

**Published:** 2022-04-28

**Authors:** Yan Zhang, Quan Wang, Wei-Kang Yang, Yong-Si Wang, Qiao Zhou, Jie Lin, Xu-Xuan Wei, Tian Liang, Tongtong Liu, Wen-Tao Fan, Li Liang, You-Nian Xu

**Affiliations:** a Department of Pathology, The First Affiliated Hospital of Guangdong University of Pharmacy, Guangzhou, China; b Department of Pathology, Maternity & Child Healthcare Hospital of Longhua District, Shenzhen, China; c College of Life Sciences and Oceanography, Shenzhen University, Shenzhen, China; d Department of Prevention and Health Care, Maternity & Child Healthcare Hospital of Longhua District, Shenzhen, China; e Guangzhou Huayin Medical Laboratory Center. Ltd, Guangzhou, China; f Department of Anesthesiology, Union Hospital, Tongji Medical College, Huazhong University of Science and Technology, Wuhan, China; g Department of Pathology, Nanfang Hospital, School of Basic Medical Sciences, Southern Medical University, Guangzhou, China; h Guangdong Province Key Laboratory of Molecular Tumor Pathology, Guangzhou, China; i Institute of Anesthesia and Critical Care Medicine, Union Hospital, Tongji Medical College, Huazhong University of Science and Technology, Wuhan, China

**Keywords:** Breast cancer, microenvironment (ME), prognosis (carcinoma), biomarker (BM), immunotherapy

## Abstract

**Purpose:** Oncology studies employing digital dissection methodologies have provided some insight on the biological features of tumor microenvironment of Triple-negative breast cancer (TNBC), but molecular diagnostics rarely have therapeutic impact. We aimed to identify a novel prognostic biomarker to investigate immune characteristics of TNBC using transcriptomic features.

**Patients and Methods:** We extracted whole transcriptome from breast cancer tissue of 30 TNBC patients and then used bioinformatics approaches to characterize the different immune cell contents in tumor tissue and para-cancerous tissue. We extract 2 indicators to describe the major differences in immune infiltration in the microenvironment between tumor tissue and para-cancerous tissue. We then combined the 2 indicators that represent the levels of increased and decreased infiltration in each sample to obtain the Immune Infiltration Score (IIS). Then we compared the tumor-infiltrating immune cell contents and immune infiltrating status in TNBC samples with CIBERSORT and ESTIMATE score to validate the IIS. Finally, 132 TNBC patients from the Cancer Genome Atlas program (TCGA) dataset was used to validate the predictive power of IIS.

**Results:** 4 types of upregulated and 4 types of downregulated immune cells were identified in the tumor tissue samples of the TNBC patients. Then we developed a novel biomarker, IIS. Results showed that IIS score can clearly separate cancer and para-cancerous tissue. Using the same cutoff value of 0 in the TNBC-TCGA cohort, we show that those patients with a higher IIS had significantly higher PD-L1 expression and shorter progression-free survival time than those with a lower IIS value, indicating IIS score can be generalized to other TNBC datasets.

**Conclusion:** we explored the immune infiltration landscape in 30 TNBC patients and provided IIS as a novel and reliable biomarker to evaluate the progression-free survival and prognosis of the TNBC patients.

## Introduction

Breast cancer is the most commonly diagnosed female cancer and comprises 23% of all cancer cases in women [1]. Changes in breast tissue composition are mediated by receptors expressed on the surface of breast cancer cells that bind chemical messengers such as hormones and impact cell fate. Hence, breast cancer types are categorised into the following groups on the basis of their receptor status: (a) oestrogen receptor (ER) or progesterone receptor (PR) positive; (b) human epidermal growth factor receptor 2 positive (amplification of cerbB2) with or without ER and PR positive; and (c) triple-negative breast cancer (TNBC) defined by the absence of ER/PR expression and human epidermal growth factor receptor 2 amplification [2]. TNBC mostly occurs in premenopausal young women under 40 years old and comprises of 15–20% of all breast cancer cases [3]. Compared with other breast cancer sub-types, TNBC survival time is shorter, and there is a higher mortality rate [3]. Due to its molecular profile, TNBC is not sensitive to endocrine or molecular targeted therapy. Consequently, surgery and chemotherapy are the main choices for systemic treatment. After the operation, the efficiency of postoperative chemo-radiotherapy is poor, and tumour recurrence is frequent [4]. Therefore, accurate identification of individuals with TNBC who are most likely to respond to therapy is an unmet clinical need.

Increasing evidence indicates that tumorigenesis is tightly associated with the immunological surveillance and defense during the disease course, and these functions play a key role in mediating response to therapy [5]. The tumour immune microenvironment (TIME) in TNBC is a highly complicated, heterogeneous construct consisting of diverse cell types and disordered gene expression. Importantly, the rapid expansion of tumour tissue induces hypoxia and necrosis, reprogramming the TIME gene expression landscape to radically affect immune cell survival, recognition, and anti-tumour function. In comparison to other breast cancers, the TIME in TNBC is associated with higher expression of vascular endothelial growth factors and other molecules promoting growth and migration of tumour cells, as well as tumour-infiltrating lymphocytes and tumour-associated macrophages [6,7]. It has been shown that the presence of tumour-infiltrating lymphocytes is correlated with a better prognosis and an improved response to neoadjuvant chemotherapy in TNBC [8–10]. Furthermore, the interaction between death receptor 1 on T cells and programmed cell death ligand 1 on tumour cells, suppresses the immune system, resulting in tumour cell immune escape [11]. As such, gaining a better understanding of the pathological impact and dynamics of different TNBC immune cells is essential for developing an effective TIME-related prognostic biomarker.

The aim of this study was to investigate immune characteristics in TNBC to identify novel prognostic biomarkers. To do this, RNA sequencing was performed in 30 TNBC patients, and bioinformatics approaches were used to differentiate the immune cell component in tumour tissue versus para-cancerous tissue. Machine learning were then used to develop a novel biomarker, termed the ‘immune infiltration score’ (IIS), which indexes the degree/presence of immune cell infiltration in the tumour microenvironment. In contrast to other available biomarkers that lack information on cellular proportions, IIS is based on the TIME immune cell component. Accordingly, IIS provides valuable information on a patient’s individual TIME composition relevant for personalised diagnosis, treatment, and prognosis.

## Methods

### Sample acquisition

30 pairs of cancerous tissue and matched para-cancerous samples from breast cancer patients who had not received chemotherapy or radiotherapy before samples collection were collected from the Maternity & Child Healthcare Hospital of Longhua District, (Shenzhen, China). The diagnosis of TNBC defined by the absence of ER/PR expression and human epidermal growth factor receptor 2 amplification was affirmed by two pathologists independently. The studies involving human participants were reviewed and approved by the Medical Ethics committee of Maternity & Child Health Care Hospital of Longhua District (NO.2019071201). All participating patients signed written informed consent and received standard-of-care treatment with surgical excision of lesions. All samples were snap-frozen using liquid nitrogen and stored at −80 °C until further processing.

### RNA extraction and cDNA reverse transcription

RNA sequencing was performed by Guangzhou Huayin Health Medical Group (Guangzhou, China). Total RNA was extracted according to the instructions of the TRIZOL kit (Invitrogen, Carlsbad, California, USA). Qualified RNA was synthesised by reverse transcription using SMARTScribe™ reverse transcriptase (Clontech, Mountain View, California, USA) to prepare 5’RACE cDNA for high-throughput sequencing. CDNA was purified using the MinElute PCR Purification Kit (Qiagen, Germany).

### Data processing and analysis

The original data obtained from high-throughput sequencing were converted to raw sequence reads by base calling, and the results were stored in FASTQ format. Low quality reads and reads without primers were discarded. PCR and sequencing errors were corrected by using unique molecular identifiers. Only duplicate reads with different unique molecular identifiers were retained in downstream processing. Original reads of RNA sequencing data were converted to normalised FPKM values. RNA-seq data have been deposited in the Annotate repository under accession numbers E-MTAB-10886.

### Immune cell quantification

Cells in TNBC and surrounding cancerous tissue samples were divided into 5 categories, epithelial cells, haematopoietic stem cells, hyaloid cells, lymphoid cells, and stromal cells (Supplementary Figure S1A–E). Epithelial cells and progenitor cells were excluded from the analysis, as they are not considered relevant to the tumour immune microenvironment. Next, the xCell method was used to quantitatively analyse cellular component differences between cancer and para-cancerous tissues [12]. xCell has the advantage/benefit to reconstruct the cellular composition by deconvolution of gene enrichment analysis profiles, and it can evaluate up to 64 cell types in a tumour environment. In addition, CIBERSORT and ESTIMATE are used to evaluate tumour-infiltrating immune cell contents and immune infiltrating status in TNBC samples to validate xCell results. We use an unsupervised clustering and visualisation method, Uniform Manifold Approximation and Projection (UMAP) algorithm in R to achieve multi-feature dimensionality reduction, and to visualise sample grouping, based on the significantly different immune cell components obtained. Z-score normalisation is used to normalise obtained cell content from the 30 tissue-normal pairs, where the mean and standard deviation is calculated by xCell score for the BRCA data set in the Cancer Genome Atlas program (TCGA).

To obtain the IIS score, we combined the 2 indicators that represented the levels of increased or decreased infiltration in each sample. In the linear combination, we define X1 and X2 to be the sum of normalised cells contents in the up-regulated and down-regulated groups, respectively. And we define a dependent variable *Y* to be a binary indicator that represents either cancer tissue or the para-cancerous tissue.

Y=aX1−bX2+c

where X1 represents the sum of cell content in the up-regulated group after normalisation, and X2 represents the sum of cell content in the down-regulated group after normalisation. Variables a and b are the linear factors related to X1 and X2, *c* represents the intercept. We used Support Vector Machine to find the best fit of the two indicators:

IIS = 1.706596 × X1 – X2 − 9.680123

Using the same methodology, we compared the best fit generated from logistic regression to the best fit derived from the Support Vector Machine, the decision boundary found by two algorithms generated very similar IIS results in TNBC-TCGA samples (cor = 0.99) (Supplementary Figure S3B). In addition, TNBC samples with IIS scores lower than 0 had significantly lower ESTIMATE scores (*p* < .01), another widely used algorithm to infer tumour infiltrating immune cells (Supplementary Figure S3A). The IIS score and corresponding X1 and X2 in our cohort are listed in Supplementary Table 3.

#### Statistical analysis

*T-*test was used to compare matched samples to find significantly altered cell content in each cell type. The Wilcoxon rank-sum test and Chi-Square test were used in comparing patient survival between TCGA IIS-positive and IIS-negative groups. Two-sided *p*-values < .05 were considered statistically significant.

## Results

### Clinicopathological characteristics

There were 30 adult patients were enrolled in this study, with 13 in grade II and 17 in grade III. Among all the patients, the age of the patients in grade II is older than those in grade III, but they were not significantly different. And the differences in tumour size, metastasis and p anthological type were not significant. The details of the clinical-pathological characteristics were listed in Tables 1 and 2.

**Table 1.** Details of the clinic pathological information of TNBC patient population.

	All patients (*n* = 30)	Grade II (*n* = 13)	Grade III (*n* = 17)	*p*
Age, years				
Mean (SD)	49.20 (9.96)	53.23 (9.18)	46.12 (9.66)	.051
Median [IQR]	48.00 [40.50, 57.00]	56.00 [47.00, 57.00]	45.00 [40.00, 52.00]	
Tuomr size, cm				
Mean (SD)	2.79 (1.46)	2.74 (1.99)	2.84 (0.96)	.861
Median [IQR]	2.50 [1.75, 3.10]	1.70 [1.70, 3.10]	2.60 [2.20, 3.10]	
Metastasis				
0	21 (70.0)	8 (61.5)	13 (76.5)	.630
1	9 (30.0)	5 (38.5)	4 (23.5)	
Pathological type				
Invasive ductal carcinoma	30 (100)	13 (100)	17 (100)	1

### Altered of immune cell infiltration in cancer tissue

**Figure 1.**
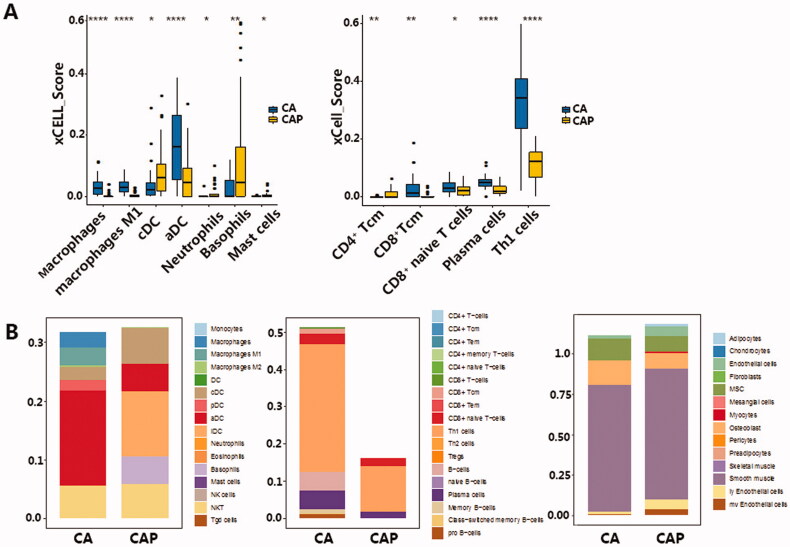
(A) Immune cells with a significant difference in cell composition between TNBC microenvironment and para-cancerous tissue. Left: Innate immune cells, Right: Adaptive immune cells. Levels of significance are represented as ns (not significant) or asterisks, *p* ≤ .05, *p* ≤ .01, *p* ≤ .001 and *p* ≤ .0001 are represented as with *, **, *** and ****respectively. aDC, activated dendritic cell; cDC, conventional dendritic cell; Tcm, central memory T cell; Tem, effector memory T cell; TH, T helper cell, CA: cancerous tissue, CAP: para-cancerous tissue. (B) Stacked bar chart of total cell content in innate immune cells (Left), adaptive immune cells (middle) and stromal cells (right). Tem, pDC, Plasmacytoid dendritic cell; lDC, lymphoid dendritic cell; NKT, Natural killer T cell; Tregs, Regulatory T cell; MSC, mesenchymal stem cell, CA: cancerous tissue, CAP: para-cancerous tissue.

**Figure 2.**
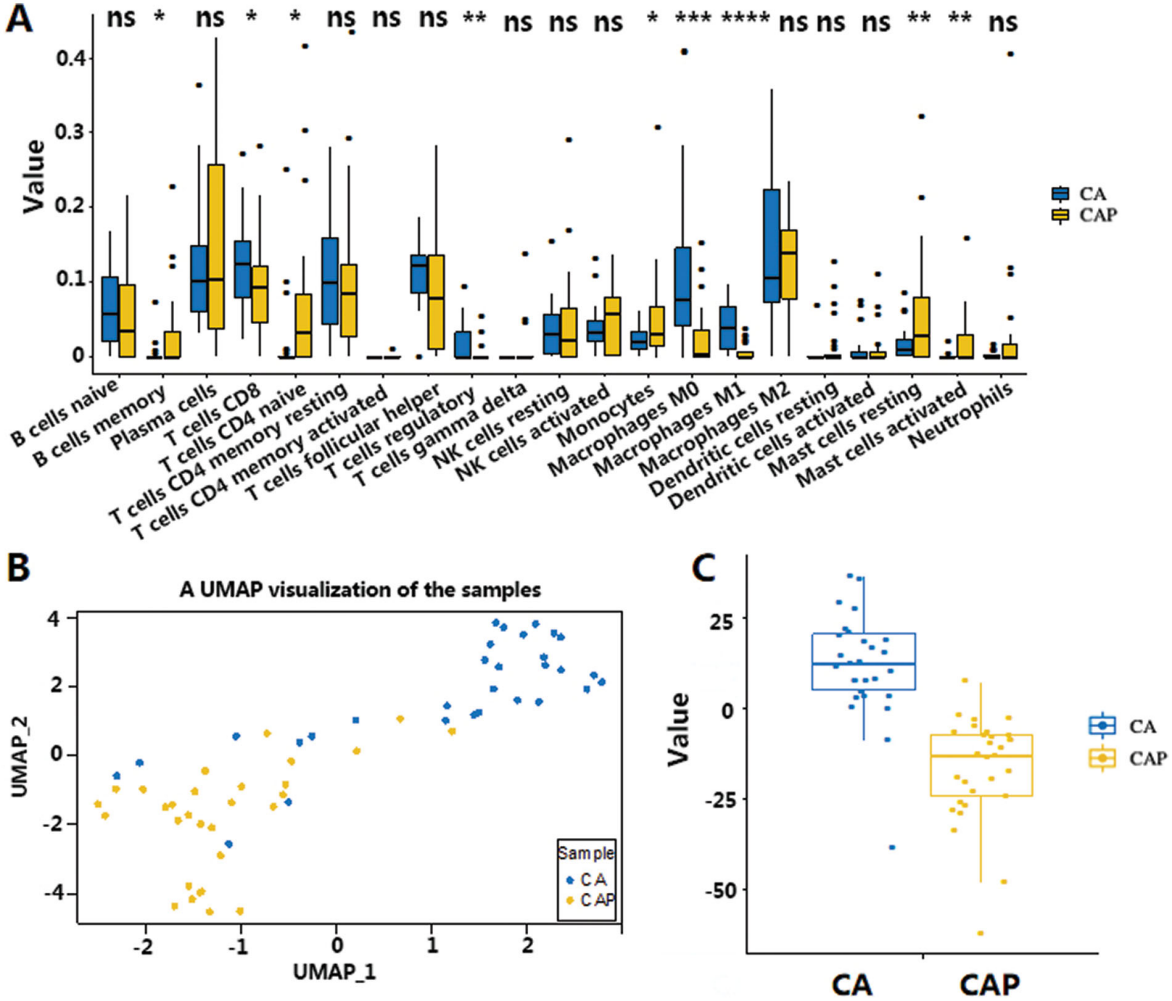
(A) Validation of cell components derived from CIBERSORT software. Macrophages, mast cells resting, and mast cells activated showed the same trend of insignificance. Although the difference in neutrophils component is not significant, it has the same trend as Xcell results. (B) UMAP clustering for 30 TNBC cancer tissues and 30 para-cancerous tissues. X axis and y axis represent UMAP components derived from immune profiles consisting of normalised immune-related cell counts in 10 types of cells, including 4 significantly up-regulated and 4 down-regulated cell components in the immune microenvironment. Levels of significance are represented as ns (not significant) or asterisks, *p* ≤ .05, *p* ≤ .01, *p* ≤ .001 and *p* ≤ .0001 are represented as with *, **, *** and ****respectively. (C) The immune infiltration score derived from the sum of normalised cells contents showed obvious differences in TNBC samples and para-cancerous samples. X axis represents TNBC and para-cancerous tissue, y axis represents the IIS score. CA: cancerous tissue, CAP: para-cancerous tissue.

We observed significant differences in the infiltration of macrophages, DC cells, granulocytes and other innate immune cells between cancer and para-cancerous tissue (Figure 1A). In TNBC, four types of immune cells were up-regulated in the TIME, including CD8 positive T cells and TH1 cells, activated dendritic cells (aDC), and macrophages (Figure 1A). The four down-regulated cell types were: Conventional dendritic cells (cDC), neutrophils, CD4 positive T cells and master cells (Figure 1A). It is worth noting that aDC showed a significant increase in the tumour tissue, in contrast to the significant decrease of cDC in tumour tissue, indicating that DC cells in the tumour microenvironment undergo a massive activation process. To validate our findings with xCell, we then used CIBERSORT to evaluate the proportion of immune cell types in the same dataset. The results in CIBERSORT showed high concordance with that in xCell (Table 3）. CD8 positive T cells, Macrophages and Mast cells are significantly changed in both xCell and CIBERSORT. Although the difference between cDC and neutrophils components is not significant, it has the same trend as xCell results (Table 2, Figure 2A).

**Table 2.** Clinicopathological baseline data.

Case	Age	Tumour size (cm)	Histology grading	No. of collected limph nodes	No. of lymph nodes with metastatic cancer	Regional lymph nodes metastasis	T stage	N stage	Operation ways
1	67	2 × 1	II	10	9	Yes	T2	N2	Radical mastectomy
2	70	NA	II	12	1	Yes	T2	N1	Radical mastectomy
3	53	1 × 3	III	12	1	Yes	T2	N1	Radical mastectomy
4	42	2.5 × 2.5 × 2	III	2	0	No	T2	N0	Radical mastectomy
5	49	1.3 × 1.7	II	16	0	No	T1	N0	Radical mastectomy
6	65	3.7 × 2.7 × 2.3	II	24	17	Yes	T2	N3	Radical mastectomy
7	46	2 × 1.3	III	15	0	No	T2	N0	Radical mastectomy
8	37	4.8 × 2.1	III	17	0	No	T2	N0	Radical mastectomy
9	36	3.6 × 1.3 × 1.5	III	37	14	Yes	T2	N3	Radical mastectomy
10	40	2.9 × 1.5 × 1.8	III	15	0	No	T2	N0	Radical mastectomy
11	66	3.1 × 1.2	II	16	1	Yes	T2	N1	Radical mastectomy
12	64	3 × 2.5 × 1.5	III	28	0	No	T2	N0	Radical mastectomy
13	46	2.5 × 1.8 × 2	III	24	0	No	T2	N0	Radical mastectomy
14	60	2.8 × 1.9	III	10	3	Yes	T2	N1	Radical mastectomy
15	59	2 × 1.9	III	11	0	No	T1	N0	Radical mastectomy
16	37	2.2 × 1.8	III	9	2	Yes	T2	N1	Radical mastectomy
17	47	2.0	III	27	0	No	T1	N0	Radical mastectomy
18	49	2.6 × 2.4	III	13	4	Yes	T2	N2	Radical mastectomy
19	57	1.7 × 1.2	II	24	8	Yes	T1	N2	Radical mastectomy
20	42	2.3 × 1.7	III	15	1	Yes	T2	N1	Radical mastectomy
21	40	1.7 × 1 × 1	II	24	0	No	T1	N0	Radical mastectomy
22	40	1.9 × 1.1	III	15	0	No	T1	N0	Radical mastectomy
23	37	5.5 × 5 × 1.2	II	20	17	Yes	T3	N3	Radical mastectomy
24	56	8 × 6 × 2	II	31	10	Yes	T3	N3	Radical mastectomy
25	43	2.5 × 2.5 × 2	III	4	0	No	T2	N0	Radical mastectomy
26	30	1.5	II	21	0	No	T1	N0	Radical mastectomy
27	66	2 × 2	II	9	1	Yes	T2	N1	Radical mastectomy
28	52	2.0	II	0	0	No	T2	N0	Breast-conserving tumour resection
29	72	7.5 × 2 × 2	III	4	0	No	T3	N0	Radical mastectomy
30	57	0.7 × 0.9	II	21	9	Yes	T1	N2	Radical mastectomy

**Table 3.** Quantitating immune cell component in xCell and Cibersort.

Cell type	xCell	Cibersort
CD8+ Tcm	Up-regulation	Up-regulation
Th1 cells	Up-regulation	Unavailable
aDC	Up-regulation	Not significant
Macrophages	Up-regulation	Up-regulation
cDC	Down-regulation	Same trend
Neutrophils	Down-regulation	Sam e trend
CD4+ Tcm	Down-regulation	Unavailable
Mast cells	Down-regulation	Down-regulation

While activation of both innate and adaptive immune cells was observed, the overall adaptive immune cell significantly increased in tumour tissue compared to the para-cancerous tissues. This increased immune infiltration in the TNBC tissue mostly comprised of elevated TH1 and B (Figure 1B). We postulate a slight decrease in the total stromal cell was also observed in TNBC samples, which may be due to this elevated immune cell component. However, we note that stromal cell levels stayed relatively stable between the two tissue types (Figure 1B). We also validated the observed activation in both innate and adaptive immune cells using the TCGA BRCA cohort. However, the TCGA BRCA cohort only showed activation in adaptive immune cells, represented by CD8+ Naive T-cells, Plasma cells and Th1 cells, no significance in the innate immune cells was observed (Figure S2A, S2B).

### The immune infiltration score

There are marked differences between tumour tissue and para-cancerous tissue. Accordingly, the study of the differential infiltration of immune cells in tumour tissue will help researchers better understand the mechanism of tumour immune surveillance. To describe the immune infiltration levels based on the changes in TNBC, the cell type content of significantly differentiated cells was extracted and used to represent the immune profile of tumour and para-cancerous tissue. Since the number of cells from each cell type can vary greatly between samples, and may likely bias the variance analysis, *Z*-score standardisation was used to ensure comparability of cell content. After that, standardised cell content from the corresponding cells was summed up to obtain 2 indicators that represented the level of increased or decreased infiltration in each sample. UMAP is used to cluster the immune profile in each sample. Figure 2B shows the separation of cancer tissue samples compared to the para-cancerous tissue samples using UMAP. The clear separation of these two groups indicates how varying levels of immune cell infiltration can have a profound impact on shaping the TNBC immune microenvironment.

**Figure 3.**
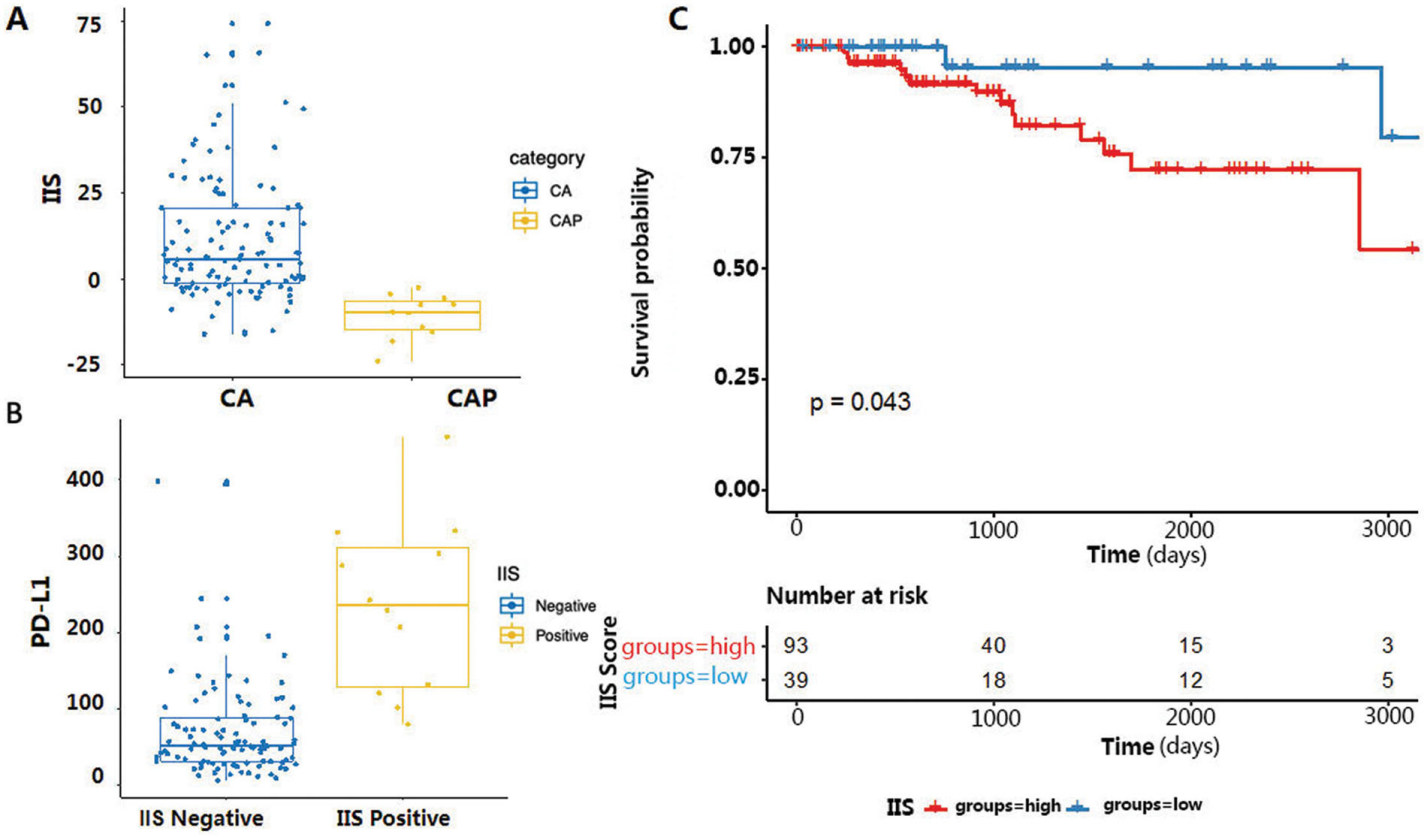
(A) Comparing IIS score in tumour and para-cancerous samples in BRCA dataset from TCGA. X-axis represents sample classification; Y-axis represents the calculated IIS score. CA: cancerous tissue, CAP: para-cancerous tissue. (B) The relationship between IIS status and PD-L1 expression in the TNBC cohort from BRCA-TCGA. X-axis represents IIS status in each sample; Y-axis represents the corresponding normalised PD-L1 RNA expression value. CA: cancerous tissue, CAP: para-cancerous tissue. (C) Kaplan Meier progression-free survival curve for 132 TNBC patients from TCGA, samples with higher IIS have a significantly shorter progression-free survival (*p* = .043).

The two indicators that represented the level of increased or decreased infiltration provide an insight into varying TIME in TNBC, we next tried to determine which linear combination of the two indicators best separated TNBC from the para-cancerous samples (Detailed training processes are described in the method section). This combination of increased and decreased infiltration in the formula *Y* = aX1-bX2 + c showed a clear separation of TNBC samples and para-cancerous samples, indicating a distinct immune infiltration state in TNBC (Figure 2C). Thus, we termed this linear combination of the increased and decreased infiltration the Immune Infiltration Score (IIS). In addition, the IIS score also showed the ability to separate cancer and para-cancerous tissue in the TNBC-TCGA cohort with the same cut-off value of 0 (Figure 3A), indicating the IIS score can be generalised to other TNBC datasets. *The correlation of IIS with TNBC survival and PD-L1 RNA expression in TNBC.*

To test if IIS had predictive power for clinical benefit in TNBC patients, we analysed the relationship of IIS score with both PD-L1 expression and the progression-free survival time with the TNBC cohort consisting of 132 identified TNBC patients from TCGA [13,14]. Results showed that patients with a higher IIS had significantly higher PD-L1 RNA expression (Figure 3B) and shorter progression-free survival time (Figure 3C) than those with a lower IIS value.

## Discussion

In recent years, multiple studies [15–17] have published methodologies for the digital dissection of tumour gene expression profiles that provide novel insight into the efficacy of breast cancer therapeutics. However, very few of these methods have been fully validated for the tumour microenvironment. Especially in TNBC, the extensive molecular heterogeneity in the tumour immune microenvironment increased the difficulty of comprehensively analyse the tumour immune microenvironment and resulted in a big stumbling block in the treatment of TNBC. In order to evaluate the immune microenvironment in TNBC, this study was first proposed to use the immune infiltration score based on the difference in cell type content between tumour tissue and the para-cancerous tissue. Furthermore, this immune infiltration score was negatively correlated with prognosis and PDL1 expression. Thus, our findings enhance the understanding of the tumour immune microenvironment in TNBC and may provide more information for the treatment of TNBC patients. All these results indicate, that IIS as an effective tool to evaluate the immune microenvironment among TNBC patients can also be used to guide the treatment of TNBC patients.

In recent years, an increasing number of studies have demonstrated that properties of the immune microenvironment are associated with the development and progression of TNBC[10]. Compared with other subtypes of BC, TNBC has a unique immune microenvironment consisting of diverse cell types and disordered gene expression. In this study, we found 4 types of immune cells were up-regulated and 4 types of immune cells were down-regulated compared to the para-cancerous tissue. This is in line with the results that tumour-associated immunological infiltrates, including dendritic cells, T cells, B cells, natural killer cells, and macrophages were important parameters of classical tumour pathology in TNBC [18,19].

We used the novel gene signature-based cell composition analysis method, xCell [12], to quantitatively analyse cell type components of tumour and para-cancerous tissue samples because xCell can investigate more immune cell types compared with Cibersort, another widely used software in cancer microenvironment research. The results analysed with Cibersort showed high concordance with that in xCell. CD8 positive T cells (Table 3), Macrophages and Mast cells are significantly changed in both xCell and Cibersort. Although the difference between cDC and neutrophils components is not significant, it has the same trend as xCell results. The results difference may be caused by the different strategies used in xCell and Cibersort. The algorithm used in xCell is based on feature gene expression, while Cibersort is on deconvolution. Considering the better performance of xCell than Cibersort using signatures, we chose xCell to quantitatively analyse cell type components of tumour and para-cancerous tissue samples and successfully developed IIS to evaluate the immune microenvironment in TNBC patients. IIS, as a binary indicator, with different values showed the different tumour immune microenvironments among TNBC patients, which may decide varied prognosis and treatment of the TNBC patients.

It is interesting to note that, when we tried to validate the predictive power of IIS in the TCGA dataset of breast cancer patients, we found higher IIS indicates poor survival probability, indicating that our IIS is well correlated with the prognosis of TNBC patients. Intriguingly, further exploration found that IIS is correlated with the PDL1 expression, which is an immune checkpoint molecule and an important target in immunotherapy in TNBC patients[20]. Therefore, IIS can guide the treatment through the variation tendency of the IIS for TNBC patients. The IIS can even throw light on the exploration of the mechanism of the immunotherapy effect differences among TNBC patients, which need further validation in the future.

## Limits

Due to the relative rarity of TNBC, the sample size of our cohort is limited. Although we do have validations in the TCGA cohort, the candidates in our cohort are all alive, the important prognosis value cannot be validated with our own data. Thus, further clinical studies are needed.

## Conclusion

In summary, we explored the immune infiltration landscape in 30 TNBC patients and compared the infiltration levels of various cell types between cancer and para-cancerous tissues. By comparing the infiltration levels of innate immune cells, adaptive immune cells, and stromal cells, we constructed an Immune Infiltration Score (IIS) from the linear combination of increased and decreased infiltrated cells in the TNBC microenvironment.

IIS is the first attempt to quantify immune activity in TNBC with our own data and further generalised to the TCGA cohort. The results those patients in the TCGA cohort with a higher IIS had significantly higher PD-L1 RNA expression and shorter progression-free survival time than those with a lower IIS value indicate IIS might be a more robust and effective tool to evaluate the immune microenvironment specifically in TNBC patients and can be used to predict the prognosis and guide the treatment. The IIS can even throw light on the exploration of the mechanism of the immunotherapy effect differences among different patients, which needs to expand the sample size and further clinical validation in the future.

## Supplementary Material

Supplemental Material

## Data Availability

The datasets used and/or analysed during the current study are available from the first author and corresponding author on reasonable request.
