# Supplementary material for: Development of an immune-related prognostic biomarker for triple-negative breast cancer
Source: Ann Med. 2022 Apr 28;54(1):1212–20. doi: 10.1080/07853890.2022.2067894 (PMC9068007; doi:10.1080/07853890.2022.2067894)
Supplement: Supplemental Material [file IANN_A_2067894_SM5245.pdf]

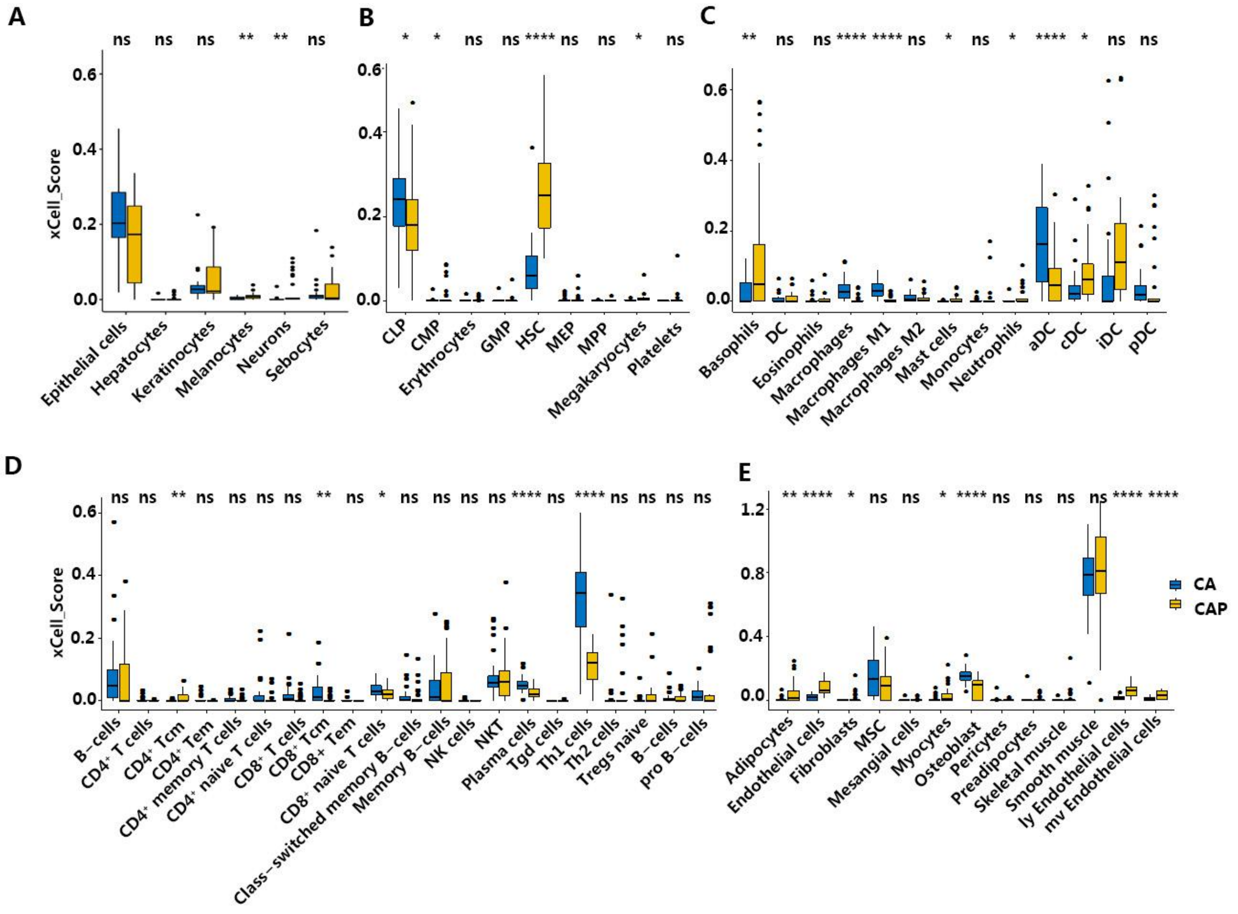

Supplementary Figure 1. Cell content for all types of cells in TNBC and peri-cancerous samples. Cells are divided into 5 categories, epithelial cells (A), Hematopoietic stem cells (B), Myeloid cells (C), Lymphoid cells (D), and Stroma cells (E). CA：cancerous tissue，CAP： peri-cancerous tissue

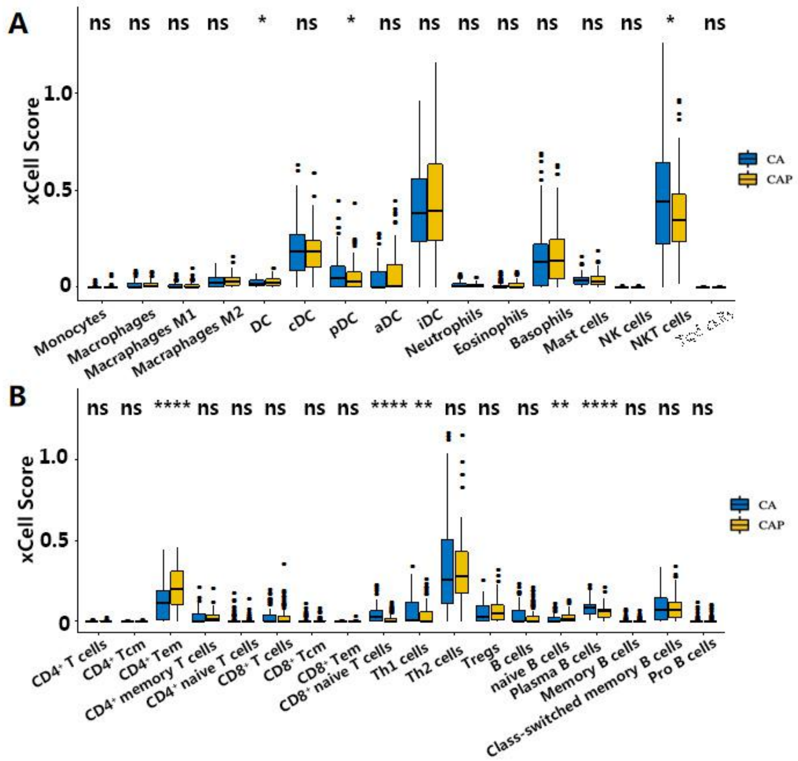

Supplementary Figure 2. Revaluation of innate immune cells (A) and adaptive immune cells (B) in TCGA's BRCA dataset using xCell. CA： cancerous tissue，CAP： peri-cancerous tissue.

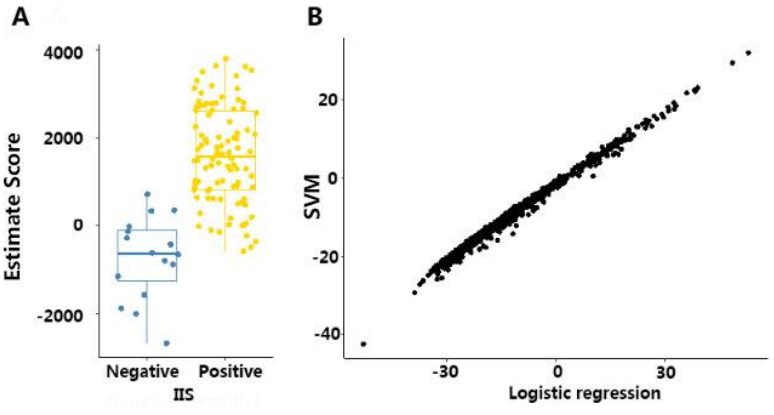

Supplementary Figure 3 (A) Comparing the Estimate score between IIS negative and IIS positive patients in 132 TNBC patients. X-axis represents IIS groups; Y-axis represents the Estimate score. (B) Correlation of SVM vs Logistic regression in 132 TNBC patients when finding the best decision boundary between cancer tissue and peri-cancerous tissue. X-axis represents IIS score generated from logistic regression; Y-axis is the corresponding IIS score generated from SVM; the latter is used to define IIS score in this paper.
